# One-Stage Versus Two-Stage Gastrectomy for Perforated Gastric Cancer: Systematic Review and Meta-Analysis

**DOI:** 10.3390/jcm14134603

**Published:** 2025-06-29

**Authors:** Michele Manara, Alberto Aiolfi, Quan Wang, Gianluca Bonitta, Galyna Shabat, Antonio Biondi, Matteo Calì, Davide Bona, Luigi Bonavina

**Affiliations:** 1 I.R.C.C.S. Ospedale Galeazzi—Sant’Ambrogio, Division of General Surgery, Department of Biomedical Science for Health, University of Milan, 20122 Milan, Italy; michele.mnra@gmail.com (M.M.); bbonit@icloud.com (G.B.); matteo.cali@unimi.it (M.C.); davide.bona@unimi.it (D.B.); 2 I.R.C.C.S. Policlinico San Donato, Division of General and Foregut Surgery, Department of Biomedical Sciences for Health, University of Milan, 20122 Milano, Italy; wangquan2013@126.com (Q.W.); galyna.shabat@gmail.com (G.S.); luigi.bonavina@unimi.it (L.B.); 3 G. Rodolico Hospital, Surgical Division, Department of General Surgery and Medical Surgical Specialties, University of Catania, 95131 Catania, Italy

**Keywords:** gastric cancer, gastric perforation, gastrectomy, damage control surgery

## Abstract

**Background/Objectives**: The optimal surgical management of perforated gastric cancer (PGC) in emergency settings remains controversial. Urgent upfront one-stage gastrectomy (1SG) and two-stage gastrectomy (2SG) with damage-control surgery followed by elective gastrectomy have been proposed. The aim of the present systematic review is to compare short- and long-term outcomes between 1SG and 2SG in the treatment of PGC. **Methods**: A systematic review and individual patient data (IPD) meta-analysis of studies reporting data of patients undergoing 1SG vs. 2SG for PGC was conducted. The time-dependent effects of surgical interventions were assessed using a likelihood ratio test. Hazard function plots were generated via marginal prediction. **Results**: Ten retrospective series (579 patients) were included. Overall, 482 patients (83%) underwent 1SG, while 97 patients (17%) were treated with 2SG. A trend toward better short-term oncological outcomes and safety profiles for 2SG compared to 1SG was observed. Long-term outcomes were comparable between 1SG and 2SG, and the IPD meta-analysis showed no statistically significant difference between the two approaches in terms of OS or hazard for mortality at all time points. A trend towards a higher hazard for mortality was observed for 1SG in the first 20 months postoperatively. **Conclusions**: Our analysis suggests that 1SG and 2SG yield comparable short-term outcomes, although 2SG may be associated with a lower medium-term mortality risk. Further research is needed to identify key factors to improve clinical judgments and decision-making in PGC.

## 1. Introduction

Gastric perforation is a common and potentially life-threatening condition, with an annual incidence of up to 14 cases per 100,000 individuals [1]. While perforated peptic ulcer remains the most frequent etiology, perforated gastric carcinoma (PGC) accounts for 10% to 16% of cases [2]. The median overall survival following urgent surgical treatment for PGC is reported to be 30.9 months [3].

The optimal surgical management of PGC remains controversial. Good clinical judgment, considering the immediate clinical emergency and the need to ensure oncological efficacy, is necessary and critical to improve patients’ outcomes [2]. Urgent upfront gastrectomy has been recommended in patients with large perforations and when the diagnosis of gastric cancer has been confirmed pre- or intraoperatively. On the other hand, damage-control surgery followed by elective gastrectomy has been proposed as a more cautious and logical approach in the attempt to perform a more radical lymphadenectomy and thereby improve survival [4,5]. Some studies suggest no significant difference in outcomes when a curative R0 resection is achieved with either approach [6]. However, more recent analyses indicate that upfront gastrectomy may compromise surgical radicality, increase morbidity, and worsen short-term outcomes compared with a two-stage approach [3]. Previous literature has provided cumulative morbidity and mortality data but only limited subgroup analyses and scarce evidence to allow rational decision-making [7,8,9].

Hence, the aim of the present systematic review is to compare short- and long-term outcomes between one-stage and two-stage gastrectomy in the treatment of PGC.

## 2. Materials and Methods

A systematic review was planned following the Preferred Reporting Items for Systematic Review and Meta-Analyses (PRISMA) guidelines [10]. The literature was searched through PubMed, MEDLINE, Scopus, Web of Science, Cochrane Central Library, Google Scholar, and ClinicalTrials.gov databases [11]. The search strategy employed multiple combinations of Medical Subject Heading (MeSH) and truncated search words, supported by Boolean operators AND/OR. The complete search strategy is reported in Appendix A.1. The research terms were perforation, perforated, gastric cancer, gastric neoplasm, stomach neoplasm, gastrectomy, complication, postoperative complication, short-term outcome, anastomotic leak, mortality, survival, long-term outcome, overall survival. Articles published up to December 1st, 2024 were considered for inclusion, along with references of relevant articles. Ethical approval was not required for this study. This study was registered on PROSPERO (CRD42024623191).

### 2.1. Eligibility Criteria

Studies reporting comparative data on short- and long-term outcomes of patients undergoing one-stage vs. two-stage gastrectomy for PGC were considered eligible for inclusion. Exclusion criteria were the following: (1) non-comparative analyses; (2) studies not reporting at least one of the predefined outcome measures; (3) not English-written; and (4) editorials and review articles.

### 2.2. Selection Process

Three independent reviewers (MM, GS, MC) performed literature reviews following the pre-determined inclusion and exclusion criteria. After the removal of duplicates, title and abstract screening was performed to detect suitable articles. A full-text review of these articles was performed to finally determine potential inclusion. Later, we searched documents that cited any of the initially included studies, as well as the references of the initially included studies to ensure the completeness of the literature review. The selection process was implemented with Rayyan Intelligent Systematic Review software. Any disagreements were addressed by two additional reviewers who were blinded to the initial assessment (DB, LB).

### 2.3. Data Collection Process

Three investigators (MM, AA, QW) independently gathered and analyzed the data, filling out predisposed tables on Google Sheets with fixed variables. The variables collected were the following: author, publication year, country, study design, study period, total number of patients, surgical procedure, age, sex, perforation site, neoadjuvant and adjuvant therapy, pathologic tumor staging, time to second-stage surgery, extent of lymphadenectomy, number of harvested lymph nodes, residual tumor classification, hospital length of stay, postoperative overall complications, anastomotic leak rate, in-hospital mortality, long-term survival. Additionally, Kaplan–Meier curves on overall survival were collected. All data were further compared at the conclusion of the review process by two authors (GB, DB) to verify and resolve inconsistencies.

### 2.4. Outcome of Interest and Definition

One-stage gastrectomy (1SG) was defined as a gastrectomy combined with lymphadenectomy performed with curative intent during the first surgical intervention. Two-stage gastrectomy (2SG) was defined as a two-step approach: the first stage addressing acute peritonitis with closure of the perforation site, followed by a second-stage curative gastrectomy with lymphadenectomy [4].

Primary outcomes were in-hospital mortality and long-term overall survival (OS). Secondary outcome measures were the number of harvested lymph nodes, anastomotic leak rate, and overall postoperative complications rate. In-hospital mortality was defined as death due to both tumor-related and non-tumor-related occurrences. OS was defined as the time from surgery to the last known follow-up and death; long-term survival data were extracted from Kaplan–Meier curves. Overall postoperative complications were defined as any deviation from the normal postoperative course requiring medical or surgical intervention, with a Clavien–Dindo grade more or equal to 2 [12].

### 2.5. Quality Assessment

The included studies’ methodological quality was judged by three independent authors (MM, AA, GB). Observational studies were evaluated applying the ROBINS-I tool, which focuses on seven specified domains: confounding bias, selection bias, classification bias, intervention bias, missing data bias, outcomes measurement bias, and reporting bias [13]. Risk of bias for each domain was graded as “Low”, “Moderate”, “Serious”, or “Critical”; overall judgment for each study was determined accordingly as of a low, moderate, serious, or critical risk of bias. The quality of the included case series was assessed according to the criteria reported by Murad et al [14]. The quality of the overall evidence from the studies was evaluated using the Grading of Recommendations, Assessment, Development, and Evaluation (GRADE) tool (https://www.gradepro.org; accessed on 31 January 2025).

### 2.6. Statistical Analysis

Data were synthesized and presented as weighted means with pooled standard deviations and weighted average percentages. A descriptive analysis was conducted, focusing on studies that reported the required data for specific outcomes, with priority given to those with a stronger methodology and more comprehensive reporting. Individual patient time-to-event data (IPD) were reconstructed from Kaplan–Meier curves using Get Data Graph Digitizer software, accessed on 19 December 2024. A flexible hazard-based regression model was developed based on IPD, incorporating a normally distributed random intercept. For periocular analyses, the baseline hazard was modeled using a degree-3 exponential B-spline without interior knots, selected according to the Akaike Information Criterion (AIC). The time-dependent effects of surgical interventions were modeled as interaction terms between surgical treatment and baseline hazard, which were assessed using a likelihood ratio test. Hazard function plots were generated via marginal prediction, with statistical significance defined as two-sided *p*-values < 0.05 and the 95% confidence intervals (CIs) reported. Statistical analyses were conducted using R software (version 3.2.2, R Foundation for Statistical Computing, Vienna, Austria).

## 3. Results

### 3.1. Systematic Review

**Figure 1:**
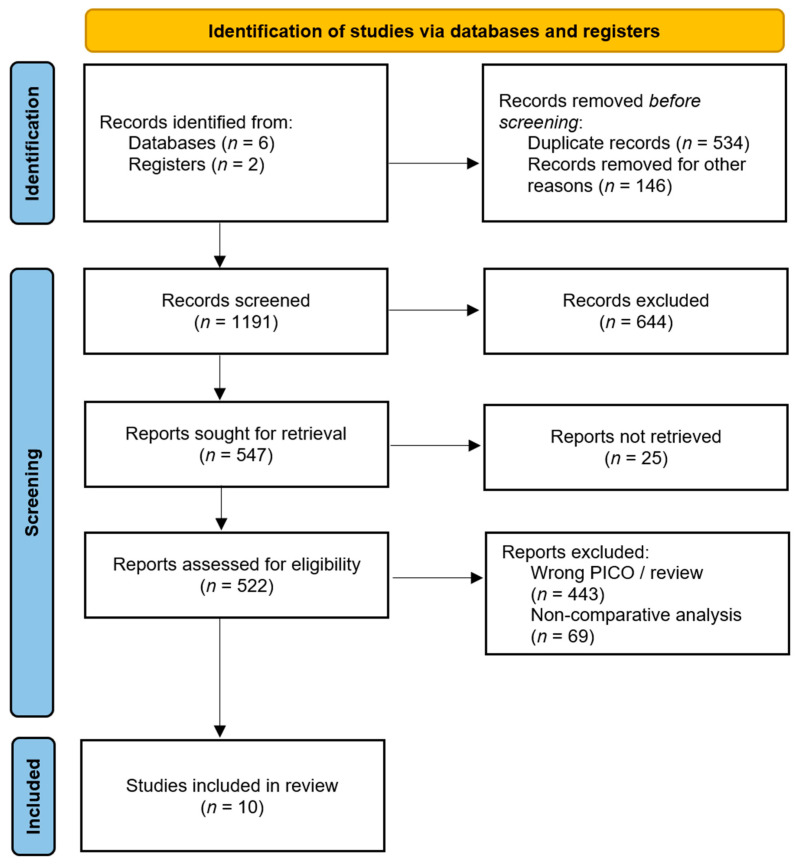
The Preferred Reporting Items for Systematic Review checklist diagram.

The PRISMA flow diagram is depicted in Figure 1. Database searching found 1871 records. After duplicates’ removal, 1191 records were screened, from which 16 documents underwent full-text evaluation. Finally, 10 papers published between 1995 and 2024 were included in the present analysis [4,5,6,15,16,17,18,19,20,21]. All the papers were retrospective cohort studies or case series. Among them, Hata et al. evaluated data from a Japanese national database of case reports from the Japan Medical Abstract Society [6]. Zhang et al. used a propensity score-matched analysis to mitigate baseline bias when comparing the two study populations [5]. The quality of the included studies is reported in Table 1.

Baseline patients’ characteristics are summarized in Table 1. A total of 579 patients with PGC treated via either 1SG (482 patients, 83%) or 2SG (97 patients, 17%) were analyzed. The age of the patients population ranged from 43.5 to 64 years [5,6,18,19], and 82% were male [4,5,17,18,19]. Pathological tumor staging was reported in five studies, with pStage 0-I, II-III, and IV accounting for 6.6%, 83.5%, and 9.9% of cases, respectively [4,5,18,19,21]. The perforation site was described in five studies, with 22.6% located in the upper third of the stomach and 77.4% in the middle-lower third [4,5,15,18,19]. Total gastrectomy was performed in 27.2% of patients, with subtotal gastrectomy in 72.8% [4,16,17,18,19]. Data on the timing between hospital admission and surgery for 1SG were limited. For 2SG, the interval between the initial and second-stage surgery, reported in four studies, ranged from approximately one week to three months [4,5,17,18]. Adjuvant treatment was documented in four studies and completed in 40.2% of subjects (29 patients) [4,15,18,20].

Operative and postoperative data are reported in Table 2. Curative resection was achieved in 77% of cases [4,5,15,16,17,18], and D2/3 lymphadenectomy was reported in 43.8% of patients [4,5,15,17,18,21]. Ozmen et al. reported no curative resections in either study group; whereas R0 resection rates in the other five studies ranged from 16 to 100% for 1SG, while it was obtained in 100% of patients submitted to 2SG [4,5,15,16,17,18]. Extensive lymphadenectomy (D2/3) was not achieved in any 1SG cases in four studies, while Kim and Zhang reported completion rates of 60–62% for 1SG. In comparison, D2/3 lymphadenectomy was accomplished in 60–100% of 2SG cases [4,5,15,17,18,21]. The number of lymph nodes retrieved was higher in 2SG compared to 1SG, as reported by Kim (33 +/− 4.3 vs. 17 +/− 3.8) and Zhang (31 +/− 2.8 vs. 17 +/− 3) [4,5].

The overall complications rate was reported in three studies and ranged from 3 to 88% for 1SG and 0 to 50% for 2SG [15,17,18]. Anastomotic leaks occurred in 16–22% of 1SG cases, with no reported leaks in 2SG [17,18]. In-hospital mortality was documented in four studies, ranging between 11 and 55% for 1SG and 0 and 50% for 2SG [6,15,16,17].

### 3.2. Meta-Analysis

**Figure 2:**
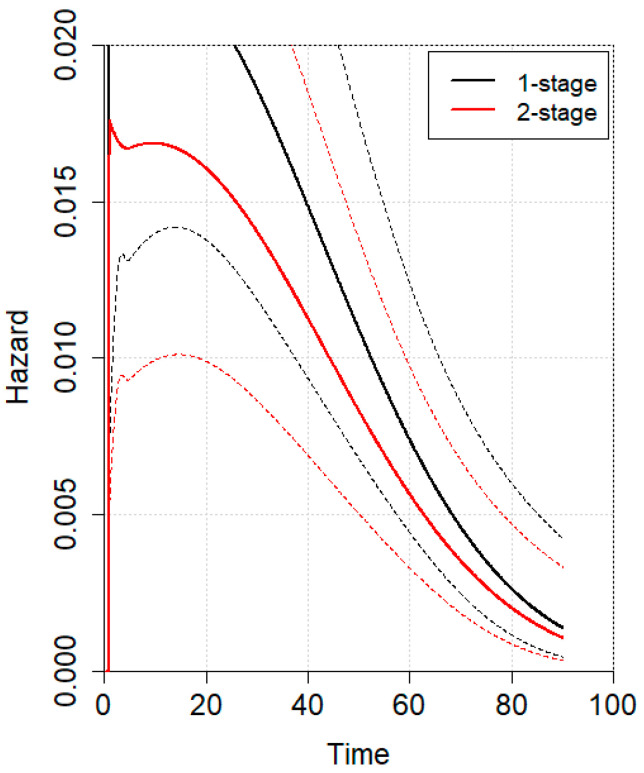
Time-varying hazard for mortality following one-stage (black line) and two-stage (red line) gastrectomy. Solid lines represent the mean estimated hazard over time, while dashed lines indicate the standard deviation for each surgical approach.

**Figure 3:**
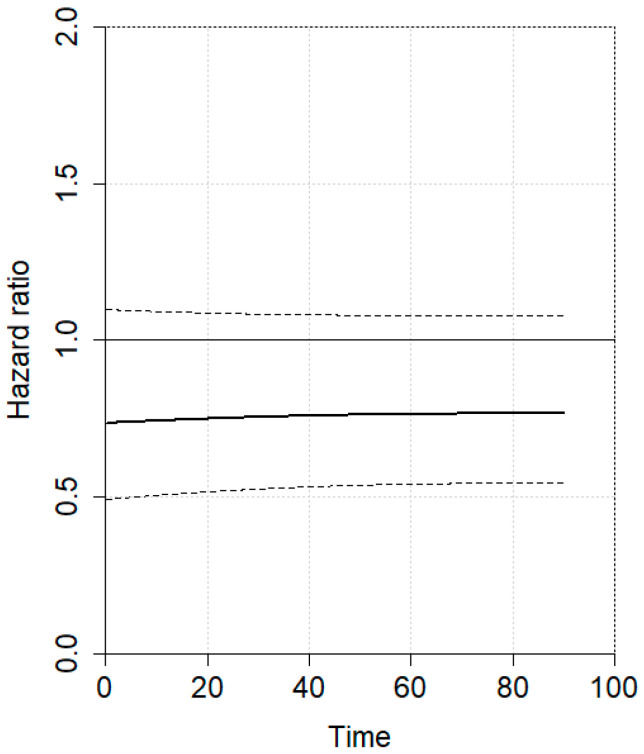
Hazard ratio for mortality between one-stage and two-stage gastrectomy. Solid line represent the mean hazard ratio over time, while dashed lines indicate the standard deviation.

**Figure 4:**
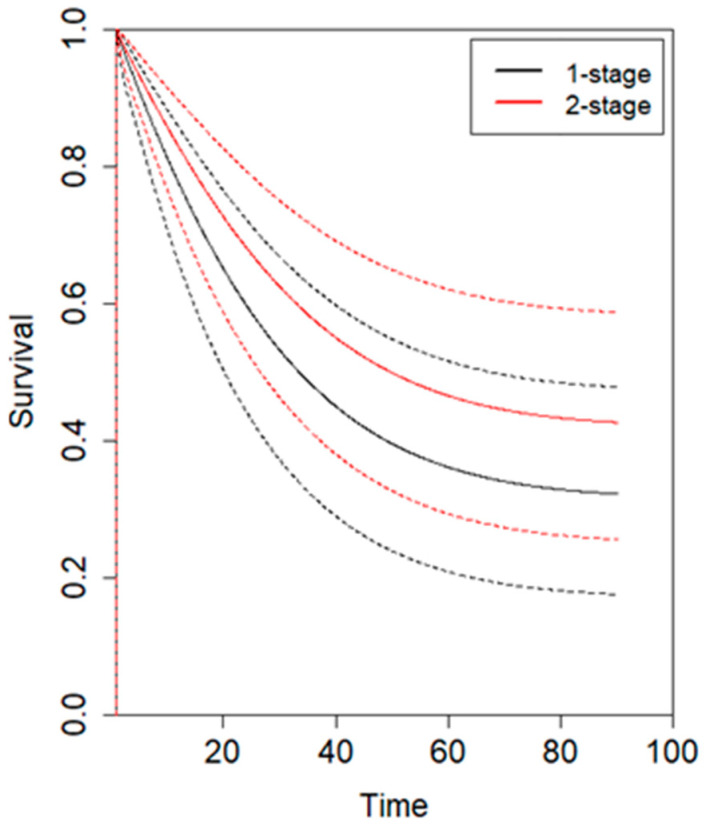
Overall survival for one-stage (black line) and two-stage (red line) gastrectomy. Solid lines represent the mean overall survival over time, while dashed lines indicate the standard deviation for each surgical approach.

An individual patient data (IPD) meta-analysis was conducted using data from four studies reporting Kaplan–Meier curves [5,6,22,23]. All included studies had a minimum follow-up of 5 years. Given the non-proportional hazard model (*p* < 0.001), the time-varying hazard for mortality in 1SG and 2SG is depicted in Figure 2. Specifically, 1SG showed a higher estimated hazard for mortality during the first 20 postoperative months, which then gradually declined at later time points compared to 2SG. No statistically significant difference was observed. The hazard ratio for mortality between 1SG and 2SG is presented in Figure 3, showing no statistically significant difference. The estimated pooled OS is illustrated in Figure 4, with a trend toward a higher survival for 2SG at all time points, though this did not reach statistical significance.

Data analysis was limited by the small sample size and the number of reported events across the studies. Consequently, a meta-analysis could not be performed for in-hospital mortality, overall complications, or anastomotic leak.

## 4. Discussion

The present analysis demonstrates comparable long-term outcomes following 1SG and 2SG for PGC. The individual patient data meta-analysis revealed no statistically significant differences in overall survival or mortality hazard between the two approaches at any time point. However, a clinical trend toward a higher mortality hazard was observed for 1SG during the first 20 postoperative months. Additionally, 2SG appeared to offer more favorable short-term oncological outcomes and a better safety profile.

Spontaneous perforation of gastric cancer is a rare complication, occurring in approximately 1% of gastric cancer patients and accounting for about 10–16% of all gastric perforations [2]. The incidence of perforation may have increased with the widespread adoption of submucosal endoscopic dissection techniques for early gastric cancer. In fact, the reported incidence of ESD-induced gastric perforation ranges from 1.2% to 6.1% [24,25]. Furthermore, the emergence of targeted therapies and immunotherapies, often combined with rescue chemotherapy for advanced tumors, may influence immune responses and metabolic stress, potentially contributing to gastrointestinal complications [26,27]. The optimal management of PGC remains a subject of debate, and the decision to pursue immediate or delayed resection is particularly challenging. Several factors may influence surgical strategy in real-world clinical practice, including a preoperatively established cancer diagnosis, the patient’s age and physiological condition, the presence of overt metastases or peritoneal carcinosis at abdominal exploration, the characteristics of the perforation, and the surgeon’s expertise.

Not knowing the diagnosis of gastric cancer at the time of initial urgent surgery may represent a critical issue in the emergency setting. In our study, a preoperative diagnosis was available in 9.1–57% of cases, and this is consistent with previous reviews [7,8,9]. Given the importance of a definitive oncological diagnosis to justify a radical surgical resection, recent publications suggest performing intraoperative endoscopy and frozen sections to rule in malignancy, especially in the case of large gastric ulcers’ perforation, non-antral ulcers, or in elderly and frail individuals [28,29,30]. However, there are concerns regarding the reliability of frozen sections in the emergency setting [7,29]. According to these data, 1SG combined with an appropriate D1-D2 lymphadenectomy should be limited to those cases where a preoperative gastric cancer diagnosis is certain.

The patient’s clinical condition and systemic inflammatory status are well-estabblished predictors for short-term postoperative complications. Several nutritional and inflammatory markers such as the neutrophil to lymphocyte ratio, Prognostic Nutritional Index (PNI), platelet to lymphocyte ratio, and Controlling Nutritional Status (CONUT) score have been developed to help predict patient outcomes [31,32,33,34,35]. In elective settings, derangements in protein and lipid metabolism, as well as immune dysfunction, can be effectively addressed to optimize preoperative performance status. However, in emergency situations, these clinical factors often dictate surgical decision-making, potentially increasing the risk of mortality and complications. A retrospective analysis of the American National Cancer Database reported a significantly higher 90-day mortality in urgent gastrectomy compared to elective resection (OR 1.30, 95% CI [1.14–1.49], *p* < 0.0001) [3]. On the contrary, a retrospective review of the American National Surgical Quality Improvement Program found no statistically significant differences in patient mortality and postoperative complications such as pneumonia or myocardial infarction [36]. In our analysis, we observed similar overall complications (3–88% vs. 0–50%) and in-hospital mortality (11–55% vs. 0–50%) rates, when comparing 1SG and 2SG. However, there was a considerable variability in postoperative morbidity across the included studies. This variability likely stems from small sample sizes in the studies reporting these outcomes, as well as the inclusion of patients with highly variable preoperative and intraoperative clinical conditions. Due to selection bias in the current literature, we were unable to perform a robust comparison between upfront 1SG and 2SG on short-term outcomes.

A critical goal in the surgical treatment of PGC, as in non-perforated gastric cancer, is to achieve a curative R0 resection with adequate lymphadenectomy [37]. The presence of an R1 margin, as determined by pathological examination, has been strongly associated with a poorer OS and 5-year OS rate in gastric cancer patients undergoing curative-intent resection [38]. While the extent of lymphadenectomy remains a topic of debate, D3 lymphadenectomy has shown similar overall survival outcomes compared to D2 lymphadenectomy in patients with resectable primary gastric carcinoma (HR 0.99, 95% CI 0.81 to 1.21). In contrast, D2 lymphadenectomy appears to offer a clinical trend toward improved overall, cancer-specific, and disease-free survival at 60-month follow-up compared to D1 dissection (95% CI −4.2, 0.7; *p* = 0.14), with a significant benefit in disease-specific survival (HR 0.81, 95% CI 0.71 to 0.92) [39,40]. When comparing 1SG and 2SG, Fisher et al. reported a lower quality of lymphadenectomy (OR 0.70, 95% CI [0.64, 0.76], *p* < 0.001) and a higher rate of positive surgical margins (OR 1.48, 95% CI [1.32, 1.65], *p* < 0.001) in urgent versus elective surgery [36]. This finding aligns with the largest study by Hata et al., which demonstrated significantly higher rates of D2 lymph node dissection (72% vs. 28.7%, *p* < 0.001) and R0 resection (78.4% vs. 50%, *p* < 0.001) in the 2SG group [6]. Similarly, in a propensity score-matched series, Zhang et al. observed a statistically significant higher number of lymph nodes harvested for 2SG compared to 1SG (31 IQR [27,38] vs. 17 IQR [12,24]; *p* = 0.009) [5]. We can therefore speculate that the decision to proceed with 1SG should be reserved for patients with an adequate physiological reserve, limited oncologic burden, and minimal or no peritoneal contamination. Delayed gastrectomy allows for patient recovery following damage-control surgery and may provide an opportunity for neoadjuvant therapy prior to curative resection. In selected cases, this approach may also facilitate the use of hyperthermic intraperitoneal chemotherapy (HIPEC) [41,42,43,44,45].

In the evolving landscape of gastric cancer treatment, the extent to which the biological characteristics of the disease influence outcomes remains poorly understood. However, ongoing research into the genetic underpinnings of gastric cancer, along with the implementation of targeted therapies and immunotherapy, holds promise for improving patient stratification and predicting outcomes more accurately in the future [46,47,48]. Emergency surgery may limit access to systemic medical therapies, thereby potentially affecting long-term prognosis. Fisher et al. reported an increased risk of all-cause mortality for urgent versus elective gastric surgeries (HR 1.21, 95% CI [1.15, 1.27], *p* < 0.0001) [3]. In our study, the reconstruction of individual patient time-to-event data from Kaplan–Meier curves was feasible in four studies [5,6,22,23]. The resulting meta-analysis revealed a clinical trend toward a lower mortality hazard for 2SG compared to 1SG during the first two postoperative years. However, no statistically significant differences were observed in 5-year overall survival or long-term mortality hazard, consistent with findings by Hata et al., who reported no significant difference in 5-year survival between 1SG and 2SG (*p* = 0.3) [6]. Several hypotheses may explain the potentially higher mortality risk during the first two years following 1SG. First, patients undergoing 1SG are often frail and unstable, with locally advanced gastric cancer requiring more extensive resections. Second, operating in a contaminated abdominal cavity could theoretically prolong the operative time and increase blood loss in patients with already diminished physiological reserves, potentially compromising or delaying the initiation of adjuvant therapy [3]. Notably, a delay of four weeks in initiating adjuvant chemotherapy has been associated with a worse OS (HR:1.05, 95% CI: 1.03–1.08, *p* < 0.001; I^2^ = 18.5%) and disease-free survival (HR:1.06, 95% CI: 1.02–1.10, *p* = 0.001; I^2^ = 40.6%). Third, peritoneal dissemination may occur in this context, as previously speculated in cases of ESD-related gastric perforation [25,49]. Positive peritoneal cytology has been significantly associated with a reduced OS (HR, 3.46; 95% CI, 2.77–4.31; *p* < 0.0001). However, in the absence of macroscopic peritoneal disease, patients with positive cytology still demonstrated better survival compared to those with overt peritoneal metastases (HR, 0.64; 95% CI, 0.56–0.73; *p* < 0.0001). Finally, 2SG may facilitate the centralization of care in high-volume centers, potentially reducing early postoperative mortality through improved failure to rescue rates [50]. Regarding tumor recurrence, Kim et al. found no significant difference in 5-year disease-free survival (52.2% vs. 80%, *p* = 0.233) between 1SG and 2SG. Although a trend toward fewer recurrences in the 2SG group was observed, it did not reach statistical significance [4]. Due to the lack of granular data, we were unable to assess the risk of oncological recurrence in detail. Prognosis is influenced by a combination of patient-related factors (e.g., gender, age, race), tumor-related characteristics (e.g., location, histology, depth of invasion, organ and peritoneal metastases), and treatment-related variables (e.g., extent of resection, lymphadenectomy) [51]. Although subgroup analyses were not feasible, our cohort exhibited substantial prognostic variability between the two surgical approaches, underscoring the need for improved patient stratification to better assess the prognostic impact of 1SG versus 2SG.

To the best of our knowledge, this is the first systematic review and meta-analysis specifically comparing 1SG and 2SG in the context of PGC. A key strength of our analysis lies in the application of individual patient data (IPD) meta-analytic methods to assess the long-term outcomes of the two procedures. However, the GRADE certainty of this evidence ranged from low to very low, necessitating caution in the interpretation of our findings. Some limitations must be acknowledged. First, the inclusion of patients with heterogeneous physiological baseline characteristics, tumor stages, and tumor localization introduce potential selection and confounding bias. Second, analyzing both curative resections and those with macroscopic or microscopic residual disease may merge two distinct prognostic scenarios. Third, the surgical data span four decades (1981–2021), introducing temporal bias due to evolving surgical techniques and perioperative systemic therapy protocols, which may affect outcomes. Fourth, data on surgeon proficiency and facility volumes were not available. Fifth, bias arising from missing data or incomplete outcome reporting (reporting bias) was observed. Since it was not possible to fully address this bias, priority was given to studies with a stronger methodology and more comprehensive reporting, allowing for a more reliable interpretation of selected outcomes within the limitations of the available evidence. Lastly, the inclusion of both total and subtotal gastrectomies, with aggregated data reporting, may confound findings due to a different operative risk profile.

## 5. Conclusions

Our analysis suggests that 1SG and 2SG yield comparable short-term outcomes, although 2SG may be associated with a lower medium-term mortality risk. Further research is needed to identify key preoperative risk factors and prognostic indicators to enhance preoperative and intraoperative risk stratification, ultimately improving clinical judgment and decision-making.

## Figures and Tables

**Table 1:** Demographic and clinical characteristics for patients undergoing one-stage versus two-stage gastrectomy for perforated gastric cancer.

Author, Contry, Year	Surgical Procedure	No. pts	Age (yrs)	M–F Ratio	Tumor Site (up–Mid/Low)	pStage 0–I	pStage II–III	pStage IV	Adjuvant Therapy	Resection (TG–STG)	QoE
Gertsch, China 1995 [15]	1SG	30	nr	nr	7–23	nr	nr	nr	0	nr	M
	2SG	2	nr	nr	0–2	nr	nr	nr	0	nr	
Lehnert, Germany 2000 [16]	1SG	2	nr	nr	nr	nr	nr	nr	nr	2–0	M
	2SG	6	nr	nr	nr	nr	nr	nr	nr	2–4	
Ozmen, Turkey 2002 [17]	1SG	9	nr	9–0	nr	nr	nr	nr	nr	4–5	S
	2SG	3	nr	3–0	nr	nr	nr	nr	nr	3–0	
Kasakura, Japan 2002 [18,22]	1SG	12	60.2 ± 14.9	10–2	8–4	2	3	7	7	3–9	M
	2SG	2	43.5 ± 5.5	2–0	0–2	1	1	0	2	0–2	
Pyrc, Germany 2006 [19]	1SG	1	70	0–1	0–1	0	1	0	nr	0–1	M
	2SG	4	52 ± 12.3	3–1	1–3	0	2	2	nr	1–3	
Kandel, Nepal 2013 [20]	1SG	5	nr	nr	nr	nr	nr	nr	5	nr	S
	2SG	1	nr	nr	nr	nr	nr	nr	1	nr	
Hata, Japan 2014 [6]	1SG	376	64 ± 11.3	nr	nr	nr	nr	nr	nr	nr	L
	2SG	54	56 ± 10.8	44–10	nr	nr	nr	nr	nr	nr	
Ignjatovic, Serbia 2016 [21]	1SG	3	nr	nr	nr	0	3	0	nr	nr	S
	2SG	5	nr	nr	nr	0	5	0	nr	nr	
Kim, Korea 2021 [23]	1SG	15	nr	14–1	1–14	3	12	0	10	1–14	M
	2SG	5	Nr	3–2	1–4	0	5	0	4	0–5	
Zhang, China 2024 [5]	1SG	29	63.7 ± 12	22–7	6–23	0	29	0	nr	nr	L
	2SG	15	62.7 ± 12.2	12–3	2–13	0	15	0	nr	nr	

One-stage gastrectomy (1SG), two-stage gastrectomy (2SG); upper third of the stomach (Up), middle or lower third of the stomach (Mid/Low); pathological tumor staging (pStage); gastrectomy type (Resection), total gastrectomy (TG), subtotal gastrectomy (STG); quality of the evidence (QoE), low–moderate–serious risk of bias (L–M–S); not reported (nr). Data are reported as numbers or mean ±standard deviation.

**Table 2:** Operative and postoperative data for patients undergoing one-stage versus two-stage gastrectomy for perforated gastric cancer.

Author, Year	Surgical Procedure	No. pts	Curative Resection	Lymphadenectomy Extension (D0/1–D2/3)	No. lymph Nodes Retrieved	Overall Complications	Anastomotic Leak	In-Hospital Mortality
Gertsch, 1995 [15]	1SG	30	23	30–0	nr	1 (3)	nr	6 (20)
	2SG	2	2	0–2	nr	0 (0)	nr	1 (50)
Lehnert, 2000 [16]	1SG	2	2	nr	nr	nr	nr	1 (50)
	2SG	6	6	nr	nr	nr	nr	0 (0)
Ozmen, 2002 [17]	1SG	9	0	9–0	nr	8 (89)	2 (22)	5 (55)
	2SG	3	0	0–3	nr	0 (0)	0 (0)	0 (0)
Kasakura, 2002 [18,22]	1SG	12	2	12–0	nr	4 (33)	2 (17)	0 (0)
	2SG	2	2	0–2	nr	1 (50)	0 (0)	0 (0)
Pyrc, 2006 [19]	1SG	1	nr	nr	nr	nr	nr	0 (0)
	2SG	4	nr	nr	nr	nr	nr	0 (0)
Kandel, 2013 [20]	1SG	5	nr	nr	nr	nr	nr	0 (0)
	2SG	1	nr	nr	nr	nr	nr	0 (0)
Hata, 2014 [6]	1SG	376	nr	nr	nr	nr	nr	42 (11)
	2SG	54	nr	nr	nr	nr	2 (4)	1 (2)
Ignjatovic, 2016 [21]	1SG	3	nr	3–0	nr	nr	nr	nr
	2SG	5	nr	2–3	nr	nr	nr	nr
Kim, 2021 [23]	1SG	15	15	6–9	17 ± 3.8	nr	nr	nr
	2SG	5	5	0–5	33 ± 4.3	0 (0)	nr	nr
Zhang, 2024 [5]	1SG	29	29	11–18	17 ± 3	nr	nr	nr
	2SG	15	15	0–15	31 ± 2.8	nr	nr	nr

One-stage gastrectomy (1SG), two-stage gastrectomy (2SG); lymphadenectomy extension according to Japanese Gastric Cancer Treatment Guidelines 2021 (6th edition); not reported (nr). Data are reported as numbers (percentage) or mean ±standard deviation.

## Data Availability

Data analyzed are available upon reasonable request from the corresponding author.

## Abbreviations

The following abbreviations are used in this manuscript:

PGC	Perforated gastric carcinoma
1SG	One-stage gastrectomy
2SG	Two-stage gastrectomy
OS	Overall survival
IPD	Individual patient time-to-event data
TG	Total gastrectomy
STG	Subtotal gastrectomy

## Appendix A

### Appendix A.1

A comprehensive literature search was conducted using multiple electronic databases.

Pubmed: (“Stomach Neoplasms”[Mesh] OR “gastric cancer”[tiab] OR “gastric neoplasm”[tiab] OR “stomach neoplasm”[tiab]) AND (“Gastrectomy”[Mesh] OR gastrectomy[tiab]) AND (“Postoperative Complications”[Mesh] OR complication[tiab] OR “postoperative complication”[tiab] OR “anastomotic leak”[tiab] OR “Treatment Outcome”[Mesh] OR “short-term outcome”[tiab] OR “long-term outcome”[tiab] OR “overall survival”[tiab] OR survival[tiab] OR mortality[tiab] OR “Perforation”[Mesh] OR perforation[tiab] OR perforated[tiab]).

MEDLINE: 1. exp Stomach Neoplasms/OR (gastric cancer OR gastric neoplasm OR stomach neoplasm).ti,ab. 2. exp Gastrectomy/OR gastrectomy.ti,ab. 3. exp Postoperative Complications/OR exp Treatment Outcome/OR complication.ti,ab. OR postoperative complication.ti,ab. OR anastomotic leak.ti,ab. 4. survival.ti,ab. OR mortality.ti,ab. OR short-term outcome.ti,ab. OR long-term outcome.ti,ab. OR overall survival.ti,ab. 5. exp Perforation/OR perforation.ti,ab. OR perforated.ti,ab. 6. 1 AND 2 AND (3 OR 4 OR 5).

Scopus: TITLE-ABS (“gastric cancer” OR “gastric neoplasm” OR “stomach neoplasm”) AND TITLE-ABS (“gastrectomy”) AND TITLE-ABS (“complication” OR “postoperative complication” OR “anastomotic leak” OR “short-term outcome” OR “long-term outcome” OR “survival” OR “overall survival” OR “mortality” OR “perforation” OR “perforated”).

Web of Science: TS = (“gastric cancer” OR “gastric neoplasm” OR “stomach neoplasm”) AND TS = (“gastrectomy”) AND TS = (“complication” OR “postoperative complication” OR “anastomotic leak” OR “short-term outcome” OR “long-term outcome” OR “survival” OR “overall survival” OR “mortality” OR “perforation” OR “perforated”)

Cochrane: (“gastric cancer” OR “gastric neoplasm” OR “stomach neoplasm”) AND (gastrectomy) AND (complication OR “postoperative complication” OR “anastomotic leak” OR “short-term outcome” OR “long-term outcome” OR survival OR “overall survival” OR mortality OR perforation OR perforated).

Google scholar: “gastric cancer” gastrectomy complication OR “postoperative complication” OR “anastomotic leak” OR mortality OR survival OR “overall survival” OR “short-term outcome” OR “long-term outcome” OR perforation.

ClinicalTrials.gov: Condition: gastric cancer. Other terms: gastrectomy, complication, anastomotic leak, survival, mortality, perforation.
